# Reperfusion-dependent treatment effects of thrombectomy in patients with large ischemic infarcts

**DOI:** 10.1177/17474930251387613

**Published:** 2025-10-07

**Authors:** Lukas Meyer, Susanne Gellißen, Götz Thomalla, Martin Bendszus, Gabriel Broocks, Matthias Bechstein, Christian Thaler, Fabien Subtil, Susanne Bonekamp, Anne H Aamodt, Blanca Fuentes, Elke R Gizewski, Michael D Hill, Antonin Krajina, Laurent Pierot, Claus Z Simonsen, Kamil Zeleňák, Rolf A Blauenfeldt, Bastian Cheng, Angélique Denis, Hannes Deutschmann, Franziska Dorn, Fabian Flottmann, Johannes C Gerber, Mayank Goyal, Jozef Haring, Christian Herweh, Silke Hopf-Jensen, Vi T Hua, Märit Jensen, Andreas Kastrup, Christiane F Keil, Andrej Klepanec, Egon Kurča, Ronni Mikkelsen, Markus Möhlenbruch, Stefan Müller-Hülsbeck, Nico Münnich, Paolo Pagano, Panagiotis Papanagiotou, Gabor C Petzold, Mirko Pham, Volker Puetz, Jan Raupach, Gernot Reimann, Peter A Ringleb, Maximilian Schell, Eckhard Schlemm, Silvia Schönenberger, Bjørn Tennøe, Christian Ulfert, Kateřina Vališ, Eva Vítková, Dominik F Vollherbst, Wolfgang Wick, Jens Fiehler, Helge Kniep

**Affiliations:** 1 Department of Diagnostic and Interventional Neuroradiology, University Medical Center Hamburg-Eppendorf, Hamburg, Germany; 2 Klinik und Poliklinik für Neurologie, Universitätsklinikum Hamburg-Eppendorf, Hamburg, Germany; 3 Department of Neuroradiology, Heidelberg University Hospital, Heidelberg, Germany; 4 Department of Neuroradiology, HELIOS Medical Center, Campus of MSH Medical School Hamburg, Schwerin, Germany; 5 Service de Biostatistique, Hospices Civils de Lyon, Lyon, France; 6 Laboratoire de Biométrie et Biologie Évolutive, Université de Lyon, Villeurbanne, France; 7 Department of Neurology, Oslo University Hospital, Oslo, Norway; 8 Norwegian University of Science and Technology, Trondheim, Norway; 9 Department of Neurology and Stroke Center, Hospital La Paz Institute for Health Research, La Paz University Hospital, Universidad Autonoma de Madrid, Madrid, Spain; 10 Department of Radiology, Medical University of Innsbruck, Innsbruck, Austria; 11 Department of Clinical Neurosciences, Hotchkiss Brain Institute, Cumming School of Medicine, University of Calgary and Foothills Medical Centre, Calgary, AB, Canada; 12 Department of Radiology, Faculty of Medicine in Hradec Kralove, Charles University, Hradec Kralove, Czech Republic; 13 Department of Neuroradiology, Hôpital Maison Blanche, Université de Reims-Champagne-Ardenne, Reims, France; 14 Department of Neurology, Aarhus University Hospital, Aarhus, Denmark; 15 Clinic of Radiology, Jessenius Faculty of Medicine, Comenius University, Martin, Slovakia; 16 Division of Neuroradiology, Vascular and Interventional Radiology, Department of Radiology, Medical University of Graz, Graz, Austria; 17 Klinik für Diagnostische und Interventionelle Neuroradiologie, Universitätsklinikum Bonn, Bonn, Germany; 18 Institute of Neuroradiology, Universitätsklinikum Carl Gustav Carus Dresden, Dresden, Germany; 19 Dresden Neurovascular Center, Universitätsklinikum Carl Gustav Carus Dresden, Dresden, Germany; 20 Department of Neurology, Faculty Hospital Trnava, Trnava, Slovakia; 21 Institut für Diagnostische und Interventionelle Radiologie und Neuroradiologie, DIAKO Krankenhaus gGmbH, Flensburg, Germany; 22 Department of Neurology, Hôpital Maison Blanche, Université de Reims-Champagne-Ardenne, Reims, France; 23 Klinik für Neurologie, Klinikum Bremen-Mitte, Bremen, Germany; 24 Institut für Neuroradiologie, Universitätsklinikum Frankfurt, Frankfurt am Main, Germany; 25 Department of Radiology, Faculty Hospital Trnava, Trnava, Slovakia; 26 Clinic of Neurology, Jessenius Faculty of Medicine, Comenius University, Martin, Slovakia; 27 Radiology Research Unit, Department of Radiology, Aarhus University Hospital, Aarhus, Denmark; 28 Klinikum Dortmund gGmbH, Klinikum der Universität Witten/Herdecke, Dortmund, Germany; 29 Klinik für Diagnostische und Interventionelle Neuroradiologie, Klinikum Bremen-Mitte, Bremen, Germany; 30 Department of Radiology, Aretaieion University Hospital, National and Kapodistrian University of Athens, Athens, Greece; 31 Vascular Neurology Research Group, German Center for Neurodegenerative Diseases (DZNE), Bonn, Germany; 32 Department of Vascular Neurology, University Hospital Bonn, Bonn, Germany; 33 Institut für Diagnostische und Interventionelle Neuroradiologie, Universitätsklinikum Würzburg, Würzburg, Germany; 34 Department of Neurology, Universitätsklinikum Carl Gustav Carus Dresden, Dresden, Germany; 35 Neurologie, Universitätsklinikum Heidelberg and Universität Heidelberg, Heidelberg, Germany; 36 Department of Neuroradiology, Oslo University Hospital, Oslo, Norway; 37 Department of Medical Imaging, St Anne’s University Hospital Brno and Faculty of Medicine, Masaryk University, Brno, Czech Republic; 38 Department of Neurology, Faculty of Medicine in Hradec Kralove, Charles University, Hradec Kralove, Czech Republic; 39 eppdata GmbH, Hamburg, Germany

**Keywords:** Stroke, thrombectomy, thrombolysis

## Abstract

**Background:**

While thrombectomy benefits patients with large infarcts, it is unclear whether this benefit persists across different levels of reperfusion.

**Aims:**

This study investigates how the degree of reperfusion influences the effectiveness of endovascular thrombectomy (EVT) combined with best medical treatment (BMT), compared to BMT alone, in patients with large infarcts.

**Methods:**

This post hoc analysis of the TENSION trial, a randomized controlled study, assessed EVT versus BMT in patients with extensive infarction (Alberta Stroke Program Early CT Score (ASPECTS) 3–5). Primary outcome was the modified Rankin Scale (mRS) score at 90 days. Secondary outcomes included infarct volume at 24 h, mortality, and symptomatic hemorrhage. Outcomes were stratified by final reperfusion level, measured with the modified thrombolysis in cerebral infarction (mTICI) scale. Confounder-adjusted common odds ratios (cORs) and average treatment effects (ATEs) were estimated using inverse probability weighting with regression adjustment.

**Results:**

A total of 246 patients (median age, 74 years (interquartile range (IQR), 65–80); median baseline ASPECTS, 4 (IQR, 3–5)) were included. Compared to BMT alone, unsuccessful EVT (mTICI ⩽ 2a) was not associated with worse functional outcomes (cOR:1.2, 95% CI, 0.95 to 1.52; p = 0.131), higher mortality (ATE: –11.6%; 95% CI, –28.82 to 5.61; p = 0.187), or larger infarct volumes on follow-up (ATE:0.99 mL; 95% CI, –45.30 to 45.32; p = 0.965). First-pass complete reperfusion (mTICI 3) showed the greatest treatment benefit, significantly improving all endpoints, with a cOR of 4.85 (95% CI, 3.74–6.31; p < 0.001) for improved mRS scores and a 29% absolute reduction in mortality.

**Conclusion:**

In this post hoc analysis of the TENSION trial, unsuccessful EVT did not worsen outcomes compared to BMT alone. The highest benefit of EVT occurred with first-pass complete reperfusion, emphasizing the importance of achieving optimal reperfusion in this vulnerable stroke subgroup. These findings do not justify general treatment recommendations.

## Graphical abstract

**Graphical abstract:**
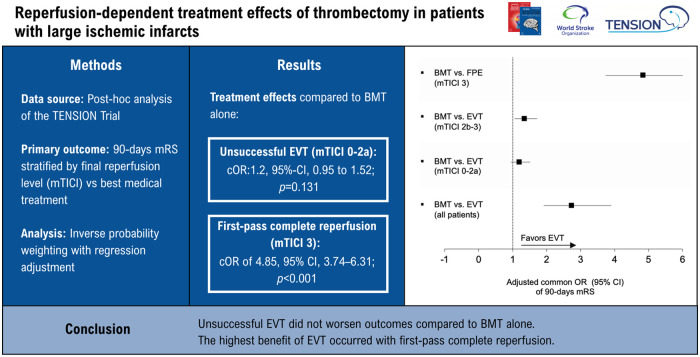

## Introduction

Evidence from randomized trials supporting efficacy and safety of endovascular thrombectomy (EVT) continues to grow, extending across multiple subgroups, including patients with large acute ischemic strokes. Treatment success of EVT is primarily attributed to reperfusion of ischemic brain due to the efficacy of mechanical thrombus removal, facilitated by advancements in modern catheter and device technology.^1–5^ Consequently, higher degrees of vessel reperfusion (i.e. successful EVT) and subsequent improved reperfusion of the affected hypoperfused arterial territory, are among the strongest procedure-related predictors of favorable long-term outcomes.^6^ Conversely, unsuccessful EVT without substantial reperfusion restoration is strongly associated with poorer clinical outcomes. Recent data from randomized trials show that EVT remains unsuccessful in approximately 20 % of cases, defined by a modified thrombolysis in cerebral infarction (mTICI) scale scores of 2a or lower at the end of the procedure.^7^

While results from the TENSION trial^2^ confirmed that patients with large infarcts benefit from EVT, extensive ischemic damage may also increase tissue vulnerability to the mechanical stresses of the procedure, potentially increasing the risk of periprocedural adverse events such as vessel perforation, dissection, and intracerebral hemorrhage. Therefore, the subgroup of stroke patients with large ischemic lesions may be more susceptible to EVT-related adverse events, particularly, in cases of unsuccessful EVT with incomplete reperfusion.^8^

We hypothesized that both treatment effects and adverse effects of EVT in the TENSION population depend on reperfusion degrees achieved, and that even lower degrees of reperfusion (i.e. unsuccessful EVT) do not result in worse outcomes compared to best medical treatment (BMT).

## Methods

### Study design and population

This study is a post hoc analysis of the TENSION (The Efficacy and Safety of Thrombectomy in Stroke with extended lesion and extended time window; NCT03094715) Trial.^2^ The TENSION Trial was an investigator-initiated, prospective, randomized, open-label, blinded endpoint (PROBE) trial conducted in Europe and Canada designed to compare treatment effects of BMT only (control group) to EVT plus BMT (intervention group) in patients with large ischemic strokes. The TENSION trial protocol and main results have been published previously.^2,9^ Reporting followed the CONSORT guideline.

### Clinical assessment

All patients underwent clinical assessments at baseline, at 24 (±6) h, at 7 days or at hospital discharge, and at 90 days (±14). Clinical assessment at 90 days was performed in-person or via telephone interview by trained personnel who were unaware of the treatment-group assignment.

### Image assessment

Imaging parameters were assessed by a central independent core laboratory (Eppdata, Hamburg, Germany). Post-treatment flow restoration following EVT was evaluated using the mTICI scale, ranging from “no” to “complete reperfusion” of the relatively hypoperfused territory distal to the occlusion site. Successful EVT was defined as mTICI 2b-3 on the final angiogram and unsuccessful EVT as no to partial reperfusion mTICI 0 to 2a. The first-pass effect was reported for mTICI 3 after the first EVT maneuver. Follow-up infarct volume was measured by independent neuroradiologists on follow-up imaging (computed tomography (CT) or magnetic resonance imaging (MRI), according to the standard of care) performed at 24 ± 8 h using semiautomatic segmentation algorithms (ITK-SNAP 3.8.0 (17) Bleeding events were considered symptomatic (sICH) in accordance with the Second European-Australasian Acute Stroke Study (ECASS II) with a clinical worsening of at least 4 points on the National Institutes of Health Stroke Scale (NIHSS) scale most likely related to hemorrhage.

### Outcome measures

Primary outcome was the functional neurological disability across the entire range of modified Rankin Scale (mRS) scores at 90 days (±14) between treatment groups in ordinal shift analysis.

Secondary outcome was independent ambulation defined as an mRS of 0 to 3 at 90 days follow-up. Imaging outcome was defined as 24 h follow-up infarct volume. Safety was evaluated by in-hospital and 90-day mortality rates, incidence of sICH, and malignant middle cerebral artery (MCA) infarction, defined as >½ MCA-territory infarct on follow-up CT with herniation requiring decompressive hemicraniectomy or causing death from edematous mass effect.

### Statistical analysis

This post hoc analysis focuses on a modified as-treated population, excluding all patients in which the primary functional endpoint was not available (Supplemental Figure S1).

Standard descriptive statistics were applied for all endpoints. Univariable distribution of metric variables was described with median and interquartile range (IQR) and categorical variables as absolute and relative frequencies.

All outcome measures were analyzed based on different degrees of reperfusion achieved through EVT and compared to control. To account for the loss of randomization and subgroup heterogeneity, treatment effects associated with varying levels of reperfusion were estimated using double-robust inverse probability weighting with regression adjustment (IPWRA), adjusting for key confounding variables, with BMT as the reference comparator.

IPWRA-based adjusted common odds ratios (cORs) and average treatment effect estimates (ATEs) were adjusted for clinically relevant confounders known to influence post-interventional outcomes and safety. These included age, sex, pre-stroke mRS status (0–2), admission NIHSS scores, baseline signs of ischemic infarction assessed via ASPECTS, administration (yes/no) of intravenous thrombolysis (IVT), and occlusion location (internal carotid artery or M1/M2 segments of the MCA). Adjusted cORs and ATEs, along with 95% confidence intervals (CIs), were calculated for selected outcome variables.

For the primary outcome, the mRS score at 90 days, adjusted cORs were reported for ordinal outcome measures. Adjusted cORs greater than 1 indicated a shift in the distribution of 90-day mRS scores toward better functional outcomes (i.e. lower mRS scores) compared to control.

For the secondary binary outcome of favorable functional status (mRS 0–3 at 90 days), ATEs were expressed as the mean increase in the probability of achieving mRS 0–3, presented in percentage points, compared to control.

ATEs for the imaging outcome, follow-up infarct volume, were reported as the mean reduction in infarct volume (measured in milliliters). For safety outcomes, ATEs were reported as the mean change in the probability of mortality and symptomatic intracranial hemorrhage (sICH), also expressed in percentage points, compared to control.

No adjustment for multiple testing was performed, and the analyses were regarded as explorative. A two-tailed p-value <0.05 was considered significant for all statistical analyses. Statistical analyses were performed with Stata/MP (version 18.0; StataCorp LLC) and R statistical software (version 4.3.2; R Project for Statistical Computing).

## Results

### Study population

A total of 246 patients (EVT, n = 126; BMT, n = 120) from the TENSION trial met the inclusion criteria and were analyzed (Supplemental Figure S1). The median age was 74 years (IQR, 65–80), 48.8% (120) were female, and the median baseline NIHSS score was 18 (IQR, 15–22). Most patients had arterial hypertension (80.6%, 191) and atrial fibrillation (33.5%, 77). The majority of occlusions were located in the M1 segment of the MCA (61.8%, 152), with a median ASPECTS of 4 (IQR, 3–5). IVT was administered in 36.2% (89) of patients.

Among EVT patients, successful reperfusion (mTICI 2b-3) was achieved in 83.3% (105), including 29.4% (37) with first-pass effect (mTICI 3 after first pass). Unsuccessful reperfusion (mTICI 0-2a) occurred in 16.7% (21; Supplemental Figure S2). Table 1 summarizes baseline characteristics by treatment and reperfusion success.

**Table 1.** Baseline characteristics stratified by reperfusion treatment levels in comparison with best medical treatment.

Baseline characteristics	BMT (n = 120)	mTICI 0-2a (n = 21)	p value^a^	mTICI 2b-3 (n = 105)	p value^a^	mTICI FPE (n = 37)	p value^a^
Age, median (IQR)	74 (66-80)	76 (66-80)	0.685	72 (65-79)	0.573	72 (67-78)	0.562
Female, % (n)	52.5 (63)	38.1 (8)	0.223	53.3 (56)	0.383	67.6 (25)	0.033
Cardiovascular risk factors, % (n)							
Atrial fibrillation	41.1 (46)	35 (7)	0.610	24.5 (24)	0.011	15.1 (5)	0.006
Arterial hypertension	80.5 (91)	71.4 (15)	0.346	82.5 (85)	0.706	75.7 (28)	0.527
Diabetes mellitus	23.7 (27)	5 (1)	0.058	27 (27)	0.577	34.3 (12)	0.212
Dyslipidemia	36.1 (39)	31.6 (6)	0.703	39.2 (38)	0.651	42.9 (15)	0.474
Coronary artery disease	22 (24)	25 (5)	0.769	28.9 (28)	0.259	27.3 (9)	0.531
Pre-stroke mRS, median (IQR)	0 (0-1)	0 (0-1)	0.280	0 (0-1)	0.171	0 (0-0)	0.045
Admission NIHSS, median (IQR)	18 (15-22)	17 (19-21)	0.549	18 (16-22)	0.648	18 (15-22)	0.899
Admission ASPECTS, median (IQR)	3 (3-4)	4 (3-4)	0.062	4 (3-5)	0.004	4 (3-5)	0.044
Occlusion location, % (n)							
ICA	35.8 (43)	42.9 (9)	0.291	33.3 (35)		29.7 (11)	
MCA M1	61.7 (74)	57.1 (12)	-	62.9 (66)	-	67.6 (25)	-
MCA M2	2.5 (3)	-	-	3.8 (4)	-	2.7 (1)	-
Intravenous thrombolysis % (n)	33.3 (40)	33.3 (7)	>0.99	40 (42)	0.3	45.9 (17)	0.163

BMT: best medical treatment; FPE: first-pass effect; IQR: interquartile range; NIHSS: National Institutes of Health Stroke Scale; ASPECTS: Alberta Stroke Program Early CT Score; mRS: modified Rankin Scale; ICA: internal carotid artery; MCA: middle cerebral artery. a: P values indicate comparisons of each reperfusion level with best medical treatment.

### Primary and secondary outcomes

**Figure 1.**
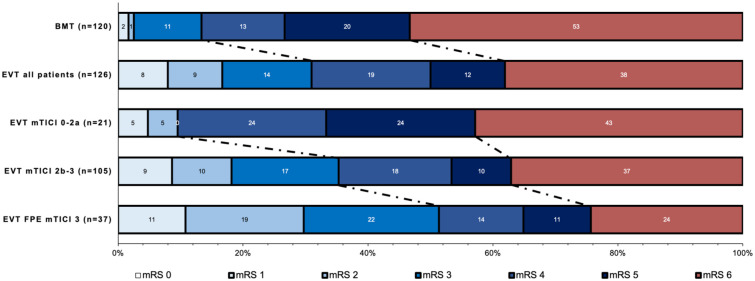
Distributions of modified Rankin Scale scores at 90 days stratified by treatment modality and reperfusion degree post-endovascular thrombectomy. BMT: best medical treatment; EVT: endovascular thrombectomy; mTICI: modified thrombolysis in cerebral infarction; mRS: modified Rankin Scale; FPE: first-pass effect.

**Figure 2.**
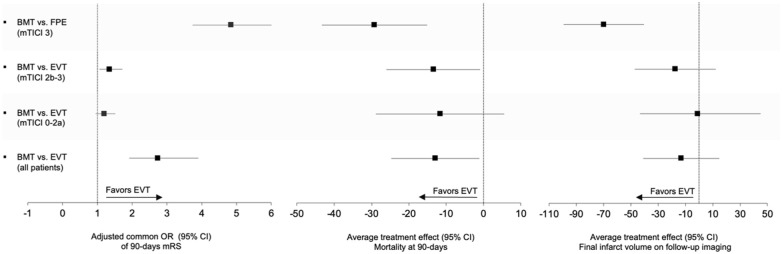
Adjusted common odds ratios and average treatment effects in the study population of functional outcome, mortality, and final infarct volume stratified by treatment modality and reperfusion degree post-endovascular thrombectomy. mTICI: modified thrombolysis in cerebral infarction; mRS: modified Rankin Scale; FPE: first-pass effect.

In univariable comparison, median functional outcomes as defined by the mRS at 90 days, were significantly better in the EVT cohort compared to the control group (EVT: 4 (IQR, 3–6), vs control: 4 (IQR, 4–6); p < 0.001; Figure 2). In addition, IPWRA estimates of adjusted cOR for improvement of mRS scores at 90 days favored EVT over control with a cOR of 2.74 (95 % CI, 1.92 to 3.90; p < 0.001). The probability for independent ambulation mRS 0–3 favored also intervention (EVT: 30.9 %, 39 vs control: 13.3 %, 16; p < 0.001, Figure 1 and Table 2).

**Table 2.** Unadjusted and adjusted outcomes stratified by treatment modality and reperfusion degree post-thrombectomy.

Outcome measures	Unadjusted analysis		p value	Adjusted analysis	p value
Functional outcome	EVT (n = 126)	BMT (n = 120)		Adjusted cOR^a^ (95% CI)	
mRS at 90 days, median (IQR)^b^	4 (3-6)	6 (4-6)	<0.001	2.74 (1.92 to 3.90)	<0.001
mTICI 3 FPE	2 (3-5)	′′	<0.001	4.85 (3.74 to 6.32)	<0.001
mTICI 2b-3	3 (4-6)	′′	<0.001	1.35 (1.06 to 1.72)	<0.013
mTICI 0-2a	5 (4-6)	′′	0.433	1.2 (0.95 to 1.52)	0.131
				Adjusted ATE^b^ (95% CI)	
mRS 0-3 at 90 days, % (n)	30.9% (39)	13.3% (16)	<0.001	17.44% (8.2 to 26.67)	<0.001
mTICI 3 FPE	51.3% (19)	′′	<0.001	38.2% (19.42 to 41.31)	<0.001
mTICI 2b-3	35.2% (37)	′′	<0.001	20.88% (11.13 to 30.63)	<0.001
mTICI 0-2a	9.5 (2)	′′	0.629	–0.7% (−15.25 to 13.74)	0.918
Imaging analysis					
Final infarct volume, ml median (IQR)	189 (93-291)	210.8 (161-293)	0.069	–14.19 (−42.21 to 13.83)	0.321
mTICI 3 FPE	128 (69-237)	′′	<0.001	–71.64 (−101.16 to –42.11)	< 0.001
mTICI 2b-3	180 (88-289)	′′	<0.001	–18.56 (−48.41 to 11.29)	0.223
mTICI 0-2a	260 (147-301)	′′	0.434	–0.99 (−45.30 to 45.32)	0.965
Safety					
Symptomatic ICH, % (n)	5.6 (7)	5 (6)	0.846	1.3% (−4.76 to 7.36)	0.674
mTICI 3 FPE	5.4 (2)	-	0.963	3.05% (−6.97 to 13.07)	0.550
mTICI 2b-3	5.7 (6)	′′	0.812	2.05% (−4.77 to 8.88)	0.555
mTICI 0-2a	4.2 (1)	′′	0.963	–0.24% (−8.83 to 8.36)	0.957
Malignant edema, % (n)	19.3 (24)	17.4 (20)	0.696	4% (−5.02 to 13.13)	0.381
mTICI 3 FPE	13.5 (5)	′′	0.799	0.52% (−15.58 to 16.63)	0.949
mTICI 2b-3	20 (21)	′′	0.864	6.2% (−3.67 to 16.08)	0.218
mTICI 0-2a	15.8 (3)	′′	1.0	–8.97% (−19.04 to 1.11)	0.081
Mortality at 90 days, % (n)	38.1 (48)	53.3 (64)	0.016	–12.9% (−24.73 to –1.07)	0.033
mTICI 3 FPE	24.3 (9)	′′	0.002	–29.24% (−43.33 to –15.15)	<0.001
mTICI 2b-3	37.1 (39)	′′	0.015	–13.46% (−25.99 to –0.93)	0.035
mTICI 0-2a	42.9 (9)	′′	0.375	–11.6% (−28.82 to 5.61)	0.187

EVT: endovascular thrombectomy; BMT: best medical treatment; CI: confidence interval; IQR: interquartile range; FPE: first-pass effect; ATE: average treatment effects; Symptomatic ICH: symptomatic intracranial hemorrhage; mTICI: modified thrombolysis in cerebral infarction; mRS: modified Rankin Scale.

a Adjusted common odds ratios derived from inverse probability weighted regression adjustment with mRS at 90 days as the dependent ordinal outcome variable.

b Average treatment effect adjusted for age, sex, pre-stroke mRS, baseline National Institutes of Health Stroke Scale (NIHSS), and Alberta Stroke Program Early CT Score (ASPECTS) on admission and occlusion location calculated with inverse probability weighting regression adjustment comparing different reperfusion levels post-thrombectomy to best medical treatment.

When stratified by reperfusion success, successful reperfusion (mTICI 2b-3) was associated with a cOR of 1.35 for a shift in the distribution of mRS scores at 90 days (95% CI, 1.06 to 1.72; p < 0.013; Figure 2 and Table 2). Highest treatment effects were observed in patients with a first-pass effect (cOR: 4.85, 95 % CI, 3.74 to 6.32; p < 0.001). Unsuccessful EVT was not associated with worse outcomes compared to control (cOR, 1.2, 95% CI, 0.95 to 1.52; p = 0.131).

### Safety analysis

Mortality rates were significantly lower in the EVT compared to control (EVT: 38.1%, 48, vs control: 53.3%, 64; p = 0.016). This effect was significant across the subgroups of successful reperfusion (ATE: –13.46 percentage points probability of mortality, 95 CI, –25.99 to –0.93; p = 0.035) and first-pass effect (ATE: –29.24%, 95 CI, –43.33 to –15.15; p < 0.001). For the EVT subgroup with unsuccessful reperfusion (mTICI 0-2a), no significant difference in mortality rates compared to control were observed. sICH occurred in seven patients (5.6%) of the EVT cohort and in six patients (5%) of the control (p = 0.846) without a significant difference of ATE (1.3%, 95 CI, –4.76 to 7.36; p = 0.674; Figure 2 and Table 2). Malignant edema occurred in 19.3% (24) of EVT patients versus 17.4% (20) of controls (p = 0.696; ATE: 4.0%, 95% CI, –5.02 to 13.13; p = 0.381), with no significant differences across subgroups (Table 2).

### Imaging analysis

Follow-up infarct volume in the EVT cohort compared to control group was numerically smaller; however, the difference was not statistically significant (189 mL vs 210.8 mL, p = 0.069; ATE –14.19 ml, 95% CI, –42.21 to 13.83; p = 0.321). For the subgroup of patients with first-pass effect, significantly smaller follow-up infarct volumes were observed (128 mL vs 210.8 mL, p < 0.001; ATE –71.64 mL, 95 CI, –101.16 to –42.11; p < 0.001; Figure 2 and Table 2).

## Discussion

This post hoc analysis of the TENSION trial revealed the following main findings for patients with large ischemic lesions at admission: (1) unsuccessful EVT did not result in worse outcomes or increased mortality compared to BMT alone; (2) The EVT-related improvement in functional outcome gradually increased in conjunction with higher degrees of reperfusion; (3) The first-pass effect, defined as mTICI 3 after one pass, led to the greatest improvement in functional outcomes; (4) substantial reductions of final infarct volumes compared to BMT were only observed in patients with a first-pass effect.

The impact of incomplete and unsuccessful EVT on functional outcomes and safety remains unclear, particularly in patients with established large infarcts. For patients with mild to moderate sings of early infarction, data from the HERMES trial showed that unsuccessful EVT in LVO stroke patients did not yield in better outcomes compared to control.^10^ In addition, unsuccessful EVT is generally associated with a higher risk of complications. These cases often involve complex occlusions requiring multiple retrieval attempts that can lead to subsequent vascular damage. Furthermore, it is often necessary to perform EVT under general anesthesia during these procedures that may introduce additional risks, including hypotension, which can exacerbate ischemic damage.^11,12^ In patients with extensive signs of infarction, the fragility of large ischemic tissue lesions may further increase the risk of procedural complications, potentially leading to worse functional outcomes in this subgroup.^8^ This raises the critical question of whether unsuccessful EVT in patients with extensive infarction might not only fail to improve outcomes but could potentially result in worse outcomes compared to control.

Our results did not reveal evidence of potential harmful treatment effects of unsuccessful EVT in patients with extensive sings of infarction at baseline. In addition, functional outcome, mortality and final infarct volumes did not differ significantly between patients with unsuccessful EVT and BMT cohort alone. This finding highlights the safety of EVT and indicates that the procedure itself should not be considered a risk factor when evaluating whether to treat patients with low ASPECTS endovascularly. Our results align with previous studies, including an analysis of data from the ESCAPE/NA-1 trial that showed similar results outside the low ASPECTS cohort.^13,14^

The analysis of treatment effects of EVT compared to control, stratified by reperfusion degree revealed a significant impact of reperfusion success on all study endpoints. Higher reperfusion degrees resulted in significant improvement in functional outcomes, favoring EVT over control. These gradually increasing effects underline the substantial benefit of achieving high reperfusion degrees, even in this subgroup with generally very poor outcome prognosis.^15^ In addition, successful reperfusion (mTICI 2b-3) was associated with a significant reduction in 90-day mortality, lowering the risk by approximately 16% compared to control. Importantly, this was achieved without an associated increase of the socioeconomically most unfavorable functional post-stroke status of mRS 5.^16^ Overall, the greatest effect sizes across all endpoints were observed when EVT was successful after the first pass (i.e. first-pass effect). This procedural surrogate is known to be a favorable predictor for outcomes in ischemic stroke patients receiving endovascular treatment.^17^ In this subgroup of patients, the first-pass effect increased the probability of independent ambulation (mRS 0-3) by 38 percentage points, while mortality decreased by 29 percentage points compared to control. Therefore, the theory of maximizing efforts using most advanced techniques and device technology for achieving mTICI 3 at the first EVT maneuver instead of achieving partial reperfusion as fast as possible seems to be the highest benchmark in the subgroup of low ASPECTS.^6,18^

Interestingly, although a trend toward gradually increasing effects in terms of infarct volume reduction was observed, a statistically significant effect on follow-up infarct volume was noted exclusively in the subgroup with first-pass effect, consistent with previous findings.^19^ However, these observed effects remain underpowered and should be interpreted with caution. This observation resumes the debate on the discrepancy between follow-up infarct volume and clinical outcome.^20,21^ The pathophysiology of large ischemic strokes, particularly in the context of acute reperfusion therapies, urgently requires further understanding.

Semi-quantitative CT-based imaging assessments may be reaching their limitations in distinguishing pan-necrosis versus incomplete infarction at the cellular level. Recent initiated discussions challenging the conventional ischemic core concept, including phenomena like selective neuronal loss and variability in tissue vulnerability, underline the complexity of these processes and highlight the need for further research.^22–24^

### Limitation

This study has all the limitations inherent in a post hoc analysis of randomized trial data. In particular, the analysis may be underpowered, as the study was not designed to compare reperfusion-dependent treatment effects. Importantly, the degree of reperfusion (study groups) was not randomized; although IPWRA was applied, residual confounding cannot be excluded, and causal inference should therefore be made with caution. Additionally, angiographic imaging is generally not included in follow-up imaging protocols; therefore, reperfusion rates and effects are unavailable in the control. In addition, there is currently a lack of a reliable gold standard for determining final infarct size on 24-h follow-up imaging.

## Conclusion

This post hoc analysis of the TENSION trial showed that unsuccessful EVT was not associated with worse outcomes or increased mortality compared to control in patients presenting with large signs of infarction. Consequently, EVT should therefore not be considered an independent risk factor in treatment decision-making. The highest benefit of EVT compared to control alone was observed when complete reperfusion was achieved at the first pass. This finding highlights the importance of prioritizing strategies and techniques aimed at achieving first-pass success as the benchmark for optimizing outcomes in this vulnerable stroke subgroup. However, because reperfusion groups were not randomized and residual confounding cannot be excluded, these results should be interpreted with caution and regarded as exploratory.

## Supplemental Material

sj-pdf-1-wso-10.1177_17474930251387613 – Supplemental material for Reperfusion-dependent treatment effects of thrombectomy in patients with large ischemic infarcts Supplemental material, sj-pdf-1-wso-10.1177_17474930251387613 for Reperfusion-dependent treatment effects of thrombectomy in patients with large ischemic infarcts by Lukas Meyer, Susanne Gellißen, Götz Thomalla, Martin Bendszus, Gabriel Broocks, Matthias Bechstein, Christian Thaler, Fabien Subtil, Susanne Bonekamp, Anne H Aamodt, Blanca Fuentes, Elke R Gizewski, Michael D Hill, Antonin Krajina, Laurent Pierot, Claus Z Simonsen, Kamil Zelenˇák, Rolf A Blauenfeldt, Bastian Cheng, Angélique Denis, Hannes Deutschmann, Franziska Dorn, Fabian Flottmann, Johannes C Gerber, Mayank Goyal, Jozef Haring, Christian Herweh, Silke Hopf-Jensen, Vi T Hua, Märit Jensen, Andreas Kastrup, Christiane F Keil, Andrej Klepanec, Egon Kurcˇa, Ronni Mikkelsen, Markus Möhlenbruch, Stefan Müller-Hülsbeck, Nico Münnich, Paolo Pagano, Panagiotis Papanagiotou, Gabor C Petzold, Mirko Pham, Volker Puetz, Jan Raupach, Gernot Reimann, Peter A Ringleb, Maximilian Schell, Eckhard Schlemm, Silvia Schönenberger, Bjørn Tennøe, Christian Ulfert, Katerˇina Vališ, Eva Vítková, Dominik F Vollherbst, Wolfgang Wick, Jens Fiehler and Helge Kniep in International Journal of Stroke
