# Supplementary material for: Reperfusion-dependent treatment effects of thrombectomy in patients with large ischemic infarcts
Source: Int J Stroke. 2025 Oct 7;21(5):646–56. doi: 10.1177/17474930251387613 (PMC13197613; doi:10.1177/17474930251387613)
Supplement: sj-pdf-1-wso-10.1177_17474930251387613 – Supplemental material for Reperfusion-dependent treatment effects of thrombectomy in patients with large ischemic infarcts [file sj-pdf-1-wso-10.1177_17474930251387613.pdf]

Supplement

Figure S1 - Flow chart of patient inclusion.

Figure S2 - Distributions of final modified thrombolysis in cerebral infarction (mTICI) scale scores.

Table S1 - Distributions of mRS scores at 90-days in the endovascular thrombectomy and best medical treatment cohort.

Table S2 - Disclosures of authors.

Table S3 - TENSION Investigator.

FIGURES

Fig S1 - Flow chart of patient inclusion.

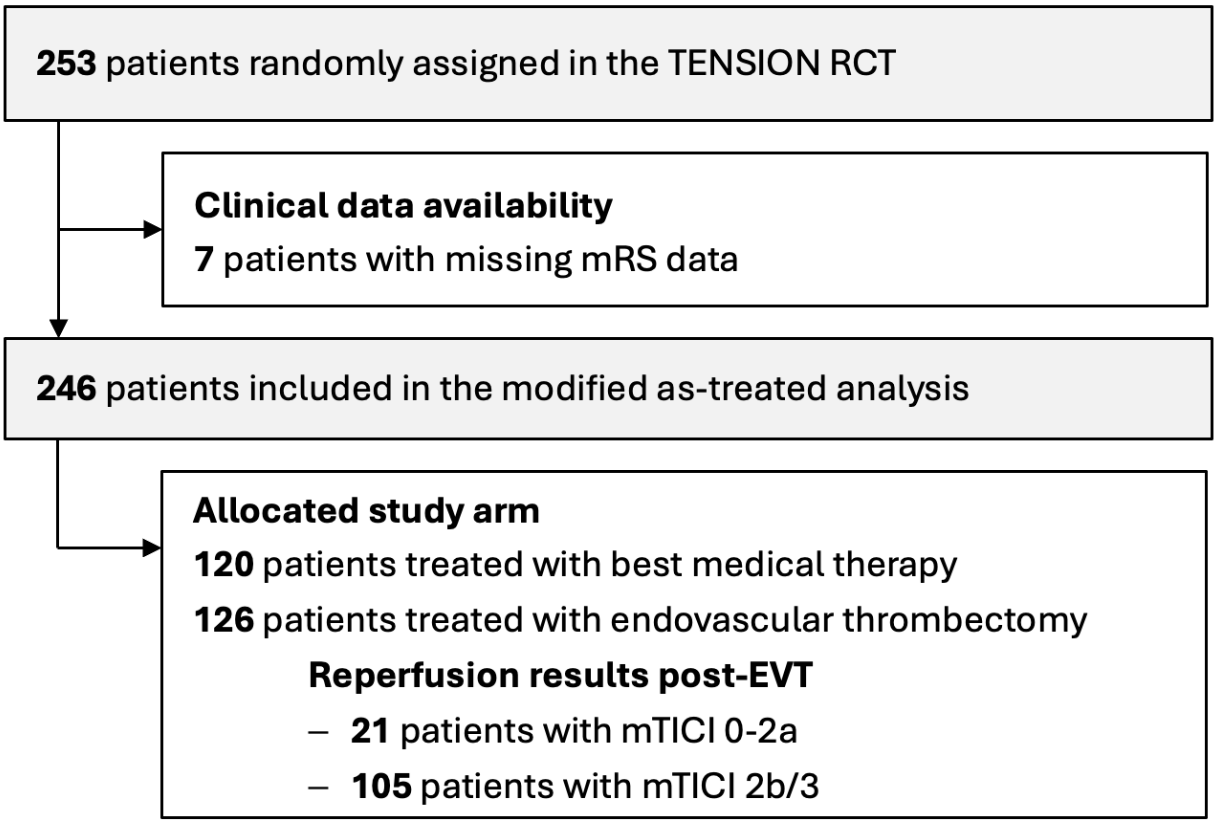

Fig S2 - Distributions of final modified thrombolysis in cerebral infarction (mTICI) scale scores.

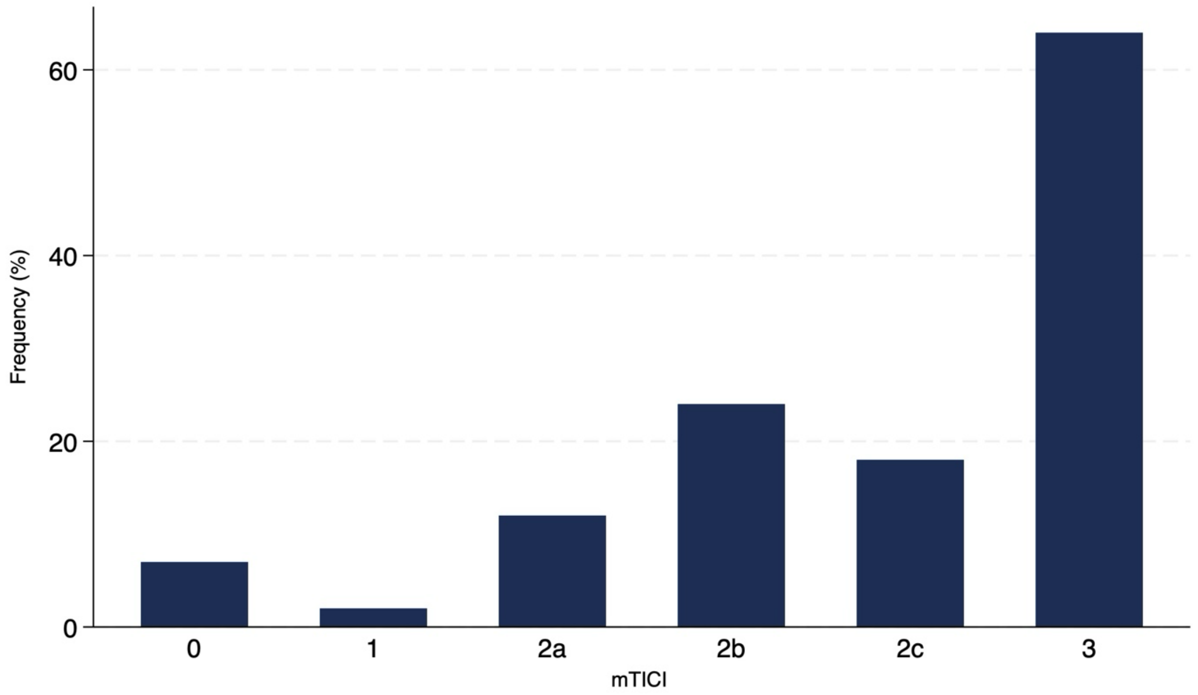

TABLES

Table S1 - Distributions of mRS scores at 90-days in the endovascular thrombectomy and best medical treatment cohort.

**mRS at 90-days**	**Endovascular thrombectomy**		**Best medical treatment**	
	n	%	n	%
1	10	7.94	2	1.67
2	11	8.73	1	0.83
3	18	14.29	13	10.83
4	24	19.05	16	13.33
5	15	11.90	24	20
6	46	38.10	66	53.33

Legend:
mRS = modified Rankin Scale

Table S2 - Disclosures of authors.

H Kniep reports compensation as speaker from Asklepios Kliniken, an ownership stake in Eppdata GmbH and compensation from Eppdata GmbH for consultant services.

L Meyer reports compensation from Eppdata GmbH for consultant services.

G Thomalla reports funding from the European Commission (EUHorizon 2020 research and innovation programme, 754640; payments to the institution); personal consulting fees from Acandis, AstraZeneca, Bayer, Boehringer Ingelheim, and Stryker; personal payment or honoraria for lectures, presentations, speakers bureaus, manuscript writing, or educational events from Acandis, Alexion, Marin, Bayer, Boehringer Ingelheim, BristolMyersSquibb/Pfizer, Daiichi Sankyo, and Stryker; participation as DSMB member for the TEA Stroke Trial (no payments) and ReSCInD trial (no payments); work as a speaker of the Commission for Cerebrovascular Diseases of the German Society of Neurology (DGN; no payments); and membership of the Board of Directors of the European Stroke Organisation (ESO; no payments).

M Jensen reports grants from the German Research Foundation and by the European Union (Horizon Europe); personal fees from Bristol Myers Squibb.

G Broocks reports compensation for consultant services from Eppdata GmbH and Acandis, and a travel grant from the American Society of Neuroradiology.

M Bendszus reports funding from EU Horizon 2020 and Deutsche Forschungsgemeinschaft (payments to the institution); honoraria for lectures from Novartis, Boehringer Ingelheim, and Seagen; and consulting fees from NeuroScios and Boehringer Ingelheim and is an editor in chief of Clinical Neuroradiology (Springer).

S Bonekamp reports funding from the EU Horizon 2020 research and innovation programme (754640 ; payments made to the institution) and EU HORIZON Research and Innovation Action 2021 (Project 101057263; payments made to the institution)

AH Aamodt reports unrestricted research grants from Boehringer Ingelheim; honoraria for lectures from BMS/ Pfizer, Teva, Roche, Abbvie, Lundbeck, and Novartis; and participation in Advisory Boards for MSD, BMS/Pfizer, Lundbeck, Lilly, and Abbvie.

B Fuentes reports research grants from Carlos III Institute of Health; personal payment for educational lectures from Servicio Madrileño de Salud; payment for lectures from Euromedice to the institution; personal payment for educational lectures from Takeda; support for attending meetings from Daiichi Sankyo; receipt of materials for research from Abbot.

MD Hill reports funding from Nil; grants to the University of Calgary for the TEMPO-2 trial from Boehringer Ingelheim, Biogen, NoNO (ESCAPE-NA1 trial and ESCAPE-NEXT trial), Canadian Institute for Health Research (ESCAPE-NA1 trial and ESCAPE-NEXT trial), Medtronic (HERMES collaboration), Alberta Innovates (QuICR Alberta Stroke Program); that some of the funds were used for the ESCAPE-NA1 trial from Alberta Innovates; consulting fees from Sun Pharma Brainsgate (paid work for adjudication of clinical trial outcomes); US patents 62/086,077 (licensed to Circle NVI) and 10,916,346 (licensed to Circle NVI); private stock ownership from Circle and PUreWeb; participation as data and safety monitoring committee chair of the RACECAT trial (end 2020), the Oncovir Hiltonel trial (ongoing), and the DUMAS trial (ongoing); participation as a data and safety monitoring committee member of the ARTESIA trial (ongoing), and the BRAIN-AF trial (ongoing); and is president of the Canadian Neurological Sciences Federation (not for profit) and a Board member of the Canadian Stroke Consortium (not for profit).

A Krajina reports grants from the European Commission for the TENSION study (payment to the institution).

L Pierot reports consulting fees from Balt, Microvention, and Phenox; and support for attending meetings or travel for the TENSION investigator meeting (transport and accommodation was reimbursed by the organization).

CZ Simonsen reports grants from Novo Nordisk Foundation and Health Research Foundation of Central Denmark Region.

RA Blauenfeldt reports speakers fees from Novo Nordisk, Pfizer and Beyer and grants from Novo Nordisk Foundation and Lundbeck Foundation.

H Deutschmann reports financial compensation for the start-up fee and the obligatory payment to the hospital administration paid by the sponsor (Medical University Heidelberg) to the clinical division (no personal payments); personal consulting fees from Stryker; speakers honorary from Medtronic; support for attending meetings or travel from Medtronic; and past presidency of the Austrian Society of Interventional Radiology and past presidency Austrian Society of Neuroradiology.

F Dorn reports consulting fees from Microvention and Balt, payment for expert testimony from Cerenovus and Microvention; payment or honoraria for lectures, presentations, speakers bureaus, manuscript writing, or educational events from Cerenovus, Stryker, Acandis, Asahi, Medtronic, Q`Apel, Tonbridge, Microvention, Inspire and Penumbra; participation in a Data Safety Monitoring Board or Advisory Board at Cerenovus and Microvention; work as an associate editor for Clinical Neuroradiology and Jouranl of Clinical Medicine and previously for Journal of NeuroInterventional Surgery.

C Herweh reports consulting fees from Brainomix and lecture fees from Stryker, Microvention Terumo and Medtronic.

S Hopf-Jensen reports funding for data collection, payment, or honoraria for lectures, presentations, speakers' bureaus, manuscript writing, or educational events from Terumo.

M Goyal reports research grants from Medtronic and Cerenovus (payments to the University of Calgary); royalties or licenses from Microvention (systems of intracranial access); personal consulting fees from Microvention, Medtronic, Stryker, Mentice, Philips, and Penumbra; and stock or stock options from Circle Neurovascular. CFK was chair of the German stroke registry (unpaid).

R Mikkelsen reports payments for a stroke lecture from TMC Academy.

M Möhlenbruch reports grants from Balt, Medtronic, MicroVention, and Stryker; consulting fees from Siemens; and support for attending meetings or travel from Europa Group.

S Müller-Hülsbeck reports consulting fees from Terumo and Boston Scientific Corporation; payment or honoraria for lectures, presentations, speakers' bureaus, manuscript writing, or educational events from Terumo and Boston Scientific Corporation.

N Münnich reports the provision of study materials.

P Pagano reports support for attending meetings or travel for the TENSION investigator meeting (transport and accommodation was reimbursed by the organisation).

M Pham reports grants from the German Research Foundation (DFG SFB 1158 A10, DFG KFO 5001 P02, DFG KFO 5001 Z, and DFG SFB TR 240 B02); speaker honoraria unrelated from Merck Serono and Bayer; and support for attending meetings or travel from Merck Serono (travel reimbursement) and Bayer (travel reimbursement).

V Puetz reports lecturer's fees from Boehringer Ingelheim, Daichii Sankyo, AstraZeneca and BMS; travel support from NoNo Inc.

PA Ringleb reports consulting fees to the institution from Boehringer Ingelheim and Bayer; and payment or honoraria for lectures, presentations, speakers' bureaus, manuscript writing, or educational events from Boehringer Ingelheim, Bayer, Pfizer, and BMS (all made to the institution).

E Schlemm reports grants from Hamburg Innovation and Hertie Foundation.

DV Vollherbst reports research grants from MicroVention; consulting fees from Medtronic; and paid lectures from Cerenovus and Johnson & Johnson.

W Wick reports consulting fees to the institution from Abbvie, BMS, GSK, and Servier.

J Fiehler reports funding from the European Commission; personal consulting fees from Acandis, Cerenovus, Medtronic, Microvention, Phenox, Stryker, and Roche; consulting at Philips (no payments); payment or honoraria for lectures, presentations, speakers bureaus, manuscript

writing or educational events from Penumbra and Tonbridge; support for attending meetings or travel from Medtronic and Penumbra; stock or stock options from Tegus Medical, Eppdata, and Vastrax; and participation in a Data Safety Monitoring Board or Advisory Board at Phenox (personal fees) and Stryker (personal fees) and is a past president of ESMINT.

F Flottmann reports consulting fees from Eppdata and support for attending meetings or travel from Microvention, Medtronic, Cerebrovascular Research and Education Foundation (CREF), and Acandis.

All other authors declare no competing interests

Table S3 – Tension Investigator.

County	Site Name	Site Investigator
Austria	Medical University Innsbruck	Prof. Dr.med. Elke R. Gizewski
Austria	Medical University Innsbruck	Prof. Dr.med. Astrid E. Grams
Austria	Medical University Innsbruck	Dr. med. Florian Dazinger
Austria	Medical University Innsbruck	Dr. med. Tanja Janjic
Austria	Medical University Innsbruck	Dr. med. Karin Gindlhuber
Austria	Medical University Innsbruck	PD Dr. med. Michael Knoflach
Austria	Medical University Innsbruck	Prof. Dr. med. Johannes Willeit
Austria	Medical University Innsbruck	Dr. med. Christian Boehme
Austria	Medical University Innsbruck	Prof. Dr.med. Stefan Kiechl
Austria	Medical University Innsbruck	Prof. Dr. med. Bernhard Glodny
Austria	Medical University Innsbruck	Ms. Karoline Volderauer
Austria	Medical University Innsbruck	Dr. med. Lukas Mayer-Süß
Austria	Medical University Innsbruck	Dr.med. Malik Galijasevic
Austria	Keppler University Hospital Linz	Dr.med. Johannes Trenkler
Austria	Keppler University Hospital Linz	Dr.med. Stephan Meckel
Austria	Keppler University Hospital Linz	Dr.med. Joachim Gruber
Austria	Keppler University Hospital Linz	Dr.med. Michael Sonnberger
Austria	Keppler University Hospital Linz	Ms. Elke Bach
Austria	Keppler University Hospital Linz	Dr.med. Eva Lenzenweger
Austria	Keppler University Hospital Linz	Dr.med. Daniel Schwarzenhofer
Austria	Medical University Graz	Prof. Dr.med. Hannes Deutschmann
Austria	Medical University Graz	Dr.med. Michael Augustin
Austria	Medical University Graz	Dr.med. Ulrike Wießpeiner
Austria	Medical University Graz	Prof. Dr.med. Rupert Horst Portugaller
Austria	Medical University Graz	Dr.med. Peter Kalmar
Austria	Medical University Graz	Dr.med. Marton Magyar
Austria	Medical University Graz	Dr.med. Eva Maria Hassler
Austria	Medical University Graz	Dr.med. Thomas Seifert-Held
Austria	Medical University Graz	Dr.med. Kurt Niederkorn
Austria	Medical University Graz	Dr.med. Thomas Gattringer
Austria	Medical University Graz	Dr.med. Alexander Pichler
Austria	Medical University Graz	Dr.med. Simon Fandler-Höfler
Austria	Medical University Graz	Siegfried Rohler
Austria	Medical University Graz	Daniela Thaler
Austria	Medical University Graz	Dr.med. Florian Schmid
Austria	Medical University Graz	Dr.med. Susanne Horner
Austria	Medical University Salzburg	Prof. Dr.med. Monika Killer-Oberpfalzer
Austria	Medical University Salzburg	Dr.med. Erasmia Müller-Thies-Broussalis
Austria	Medical University Salzburg	Dr.med. Sebastian Mutzenbach
Austria	Medical University Salzburg	Dr.med. Slaven Pikija
Austria	Medical University Salzburg	Dr.med. Constantin Hecker
Austria	Medical University Salzburg	Dr.med. Rahman Al-Schameri
Austria	Medical University Salzburg	Dr.med. Manuel Lunzer
Austria	Medical University Salzburg	Dr.med. Michael Kral
Austria	Medical University Salzburg	Dr.med. Friedrich Weymayr
Austria	Medical University Salzburg	Dr.med. Nele Bubel
Austria	Medical University Salzburg	Dr.med. Bernhard Ganser
Austria	Medical University Salzburg	Dr.med. Ursula Leitner
Austria	Medical University Salzburg	Dr.med. Floriana Hacker-Ivan
Austria	Medical University Salzburg	Dr.med. Tobias Moser
Austria	Medical University Salzburg	Dr.med. Rudolf Kreidenhuber
Austria	Medical University Salzburg	Dr.med. Asima Delalic
Austria	Medical University Salzburg	Dr.med. Markus Leitinger
Austria	Medical University Salzburg	Prof. Dr.med. Pfaff Johannes
Austria	Medical University Salzburg	Dr. Kamila Volna
Austria	Medical University Salzburg	Dr. Ferdinand Otto

Austria	Medical University Salzburg	Dr. Matthias Mauritz
Austria	Medical University Salzburg	Angela Jedlitschka

Canada	University of Calgary	Michael D. Hill, MD. MSc FRCPC
Canada	University of Calgary	Mayank Goyal, MD. PhD.
Canada	University of Calgary	Karla J Ryckborst, RN
Czech Republic	Faculty Hospital Hradec Kralove	Prof. Antonín Krajina
Czech Republic	Faculty Hospital Hradec Kralove	MD PhD Vendelín Chovanec
Czech Republic	Faculty Hospital Hradec Kralove	Assoc. Prof. Jan Raupach
Czech Republic	Faculty Hospital Hradec Kralove	MD Eva Vítková
Czech Republic	Faculty Hospital Hradec Kralove	MD Oldřich Medek
Czech Republic	Faculty Hospital Hradec Kralove	MD PhD Ondřej Renc
Czech Republic	Faculty Hospital Hradec Kralove	MD David Matyáš
Czech Republic	St. Anne´s University Hospital Brno	MD Kateřina Vališ
Czech Republic	St. Anne´s University Hospital Brno	MD Tomáš Křivka
Czech Republic	St. Anne´s University Hospital Brno	MD Igor Suškevič
Czech Republic	St. Anne´s University Hospital Brno	Assoc. Prof. Jiří Vaníček
Czech Republic	St. Anne´s University Hospital Brno	MD Bohuslav Vojtíšek
Czech Republic	St. Anne´s University Hospital Brno	Prof. Robert Mikulík
Czech Republic	St. Anne´s University Hospital Brno	D PhD Stanislava Jakubíček
Czech Republic	St. Anne´s University Hospital Brno	MD PhD Ilona Eliášová
Czech Republic	St. Anne´s University Hospital Brno	MD Viktor Weiss
Czech Republic	St. Anne´s University Hospital Brno	MD David Goldemund
Czech Republic	St. Anne´s University Hospital Brno	MD PhD Ondřej Strýček
Czech Republic	St. Anne´s University Hospital Brno	MD Jiří Búřil
Czech Republic	St. Anne´s University Hospital Brno	MD PhD Michal Haršány
Czech Republic	St. Anne´s University Hospital Brno	MD PhD Irena Doležalová
Czech Republic	University Hospital Ostrava	MD PhD Martin Roubec
Czech Republic	University Hospital Ostrava	MD Eva Hurtíková
Czech Republic	University Hospital Ostrava	Prof. Michal Bar
Czech Republic	University Hospital Ostrava	Assoc. Prof. Ondřej Volný
Czech Republic	University Hospital Ostrava	MD PhD Tomáš Jonszta
Czech Republic	University Hospital Ostrava	MD Martin Čábal
Czech Republic	University Hospital Ostrava	MD Richard Novobilský

Czech Republic	University Hospital Ostrava	Petra Brodová
Czech Republic	University Hospital Ostrava	MSc Nina Čurdová
Czech Republic	Homolka Hospital Prague	MD Martin Kovář
Czech Republic	Homolka Hospital Prague	MD Michal Panský
Czech Republic	Homolka Hospital Prague	MD Pavel Mencl
Czech Republic	Homolka Hospital Prague	MD Alena Šnajdrová
Czech Republic	Homolka Hospital Prague	Milada Roztočilová
Denmark	Aarhus University Hospital	MD PhD Claus Ziegler Simonsen
Denmark	Aarhus University Hospital	MD DMSc Grethe Andersen
Denmark	Aarhus University Hospital	MD PhD Marie Louise Schmitz
Denmark	Aarhus University Hospital	MD PhD Dorte Damgaard
Denmark	Aarhus University Hospital	MD PhD Niels Hjort
Denmark	Aarhus University Hospital	MD Marika Poulsen
Denmark	Aarhus University Hospital	MD Tove Diedrichsen
Denmark	Aarhus University Hospital	MD PhD Kristina DupontHougaard
Denmark	Aarhus University Hospital	MD PhD Poul VonWeitzel-Mudersbach
Denmark	Aarhus University Hospital	MD Rolf AnkerlundBlaufeldt
Denmark	Aarhus University Hospital	MD Sanja Karabegovic
Denmark	Aarhus University Hospital	MD Lasse ZachoSpeiser
Denmark	Aarhus University Hospital	MD Leif HougaardSoerensen
Denmark	Aarhus University Hospital	Rikke BayThomsen
Denmark	Aarhus University Hospital	Schiela Jensen
Denmark	Aalborg University Hospital	Dr. Boris Modrau
Denmark	Aalborg University Hospital	Dr. Krystian Figlewski
Denmark	Aalborg University Hospital	Maiken Falkesgaard
Denmark	Aalborg University Hospital	Nina HjortJensen
Denmark	Aalborg University Hospital	Dr. Jan PlougmannPovlsen
Denmark	Aalborg University Hospital	Dr. Agnieszka Delekta
Denmark	Aalborg University Hospital	Dr. Maria Theresa Simonsen
Denmark	Aalborg University Hospital	Dr. Joanna Oder
Denmark	Aalborg University Hospital	Dr. Svetlana Rudnicka
Denmark	Aalborg University Hospital	Dr. Fadha Elawi Al-Kuzae
France	CHU Reims Hôpital Maison Blanche	Pr Laurent Pierot
France	CHU Reims Hôpital Maison Blanche	Dr. Paolo Pagano
France	CHU Reims Hôpital Maison Blanche	Dr. Vi Tuan Hua
France	Hôpitaux Universitaires Paris – Pitié-Salpêtrière	Prof Fréderic Clarençon
France	Hôpitaux Universitaires Paris – Pitié-Salpêtrière	Dr. Nader Sourour
France	Hôpitaux Universitaires Paris – Pitié-Salpêtrière	Dr. Eimad Shotar
France	Hôpitaux Universitaires Paris – Pitié-Salpêtrière	Dr. Stéphanie Lenck
France	Hôpitaux Universitaires Paris – Pitié-Salpêtrière	Dr. Kévin Premat
France	Hôpitaux Universitaires Paris – Pitié-Salpêtrière	Dr. Julien Allard
France	Hôpitaux Universitaires Paris – Pitié-Salpêtrière	Pr Charlotte Rosso

France	Hôpitaux Universitaires Paris – Pitié-Salpêtrière	Dr. Sophie Crozier
France	Hôpitaux Universitaires Paris – Pitié-Salpêtrière	Dr. Sara Leder
France	Hôpitaux Universitaires Paris – Pitié-Salpêtrière	Dr. Anne Leger
France	Hôpitaux Universitaires Paris – Pitié-Salpêtrière	Dr. Christine Vassilev
France	Hôpitaux Universitaires Paris – Pitié-Salpêtrière	Dr. Stephen Delorme
France	Hôpitaux Universitaires Paris – Pitié-Salpêtrière	Dr. Flore Baronnet
France	Hôpitaux Universitaires Paris – Pitié-Salpêtrière	Dr. Aymeric Wittwer
France	CHU de Rennes	Prof. Jean-Christophe Ferré
France	CHU de Rennes	Dr. Quentin Alias
France	CHU Gabriel Montpied, Clermont-Ferrand	Dr. Emmanuel Chabert
France	CHU Gabriel Montpied, Clermont-Ferrand	Docteur Anna Ferrier
Germany	University Hospital Heidelberg	Prof. Dr. Martin Bendszus
Germany	University Hospital Heidelberg	Prof Dr. Markus A. Möhlenbruch
Germany	University Hospital Heidelberg	PD Dr. Fatih Seker
Germany	University Hospital Heidelberg	Christian Ulfert
Germany	University Hospital Heidelberg	Dr. Alexander Mohr
Germany	University Hospital Heidelberg	PD Dr. Michael Breckwoldt
Germany	University Hospital Heidelberg	PD Dr. Ulf Neuberger
Germany	University Hospital Heidelberg	PD Dr. Dominik Vollherbst
Germany	University Hospital Heidelberg	Dr. Christian Herweh
Germany	University Hospital Heidelberg	Dr. Jessica Jesser
Germany	University Hospital Heidelberg	Dr. Leonie Jestaedt
Germany	University Hospital Heidelberg	PD Dr. Moritz Kronlage
Germany	University Hospital Heidelberg	Dr. Daniel Schwarz
Germany	University Hospital Heidelberg	Prof Dr. Peter Arthur Ringleb
Germany	University Hospital Heidelberg	Prof Dr. Jan Purrucker
Germany	University Hospital Heidelberg	Prof. Dr. Silvia Schönenberger
Germany	University Medical Center Hamburg	Prof. Dr. med. Casper Brekenfeld
Germany	University Medical Center Hamburg	Prof. Dr. med. Götz Thomalla
Germany	University Medical Center Hamburg	Prof. Dr. med. Jens Fiehler
Germany	University Medical Center Hamburg	Dr. med. Julia Hoppe
Germany	University Medical Center Hamburg	PD Dr. med. Bastian Cheng
Germany	University Medical Center Hamburg	Dr. med. Milani Deb-Chatterji
Germany	University Medical Center Hamburg	PD Dr. med. Gellißen Susanne
Germany	University Medical Center Hamburg	Dr. med. Eckhard Schlemm
Germany	University Hospital Bochum	Prof. Dr. med. Werner Weber
Germany	University Hospital Bochum	PD Dr. med. Sebastian Fischer
Germany	University Hospital Bochum	Dr. med. Anushe Weber
Germany	University Hospital Bochum	Dr. med. Andreas Oldag
Germany	Dortmund Klinikum Mitte	Prof. Dr. Stefan Rohde
Germany	Dortmund Klinikum Mitte	Dr. Olaf Adamczewski
Germany	Dortmund Klinikum Mitte	Dr. Tobias Breyer

Germany	Klinikum Stuttgart – Katharinenhospital	Dr. med. Victoria Hellstern
Germany	Klinikum Stuttgart – Katharinenhospital	Prof. Dr. med. Hans Henkes
Germany	Klinikum Rechts der Isar Technische Universität München	PD Dr. Christian Maegerlein
Germany	Klinikum Rechts der Isar Technische Universität München	Dr.med. Silke Wunderlich
Germany	Klinikum der Universität München	Univ.-Prof. Dr.med. Thomas Liebig
Germany	Klinikum der Universität München	PD Dr.med. Franziska Dorn
Germany	Klinikum der Universität München	Dr. med. Christian Brem
Germany	Klinikum der Universität München	Dr. med. Robert Forbig
Germany	Klinikum der Universität München	PD Dr. Christoph Trumm
Germany	Klinikum der Universität München	Dr. med. Lars Kellert
Germany	Klinikum der Universität München	Dr. med. Frank Wollenweber
Germany	Universitätsklinikum Frankfurt	Prof. Dr. med. Richard du Mesnil de Rochemont
Germany	Universitätsklinikum Frankfurt	Prof. Dr. med. Marlies Wagner
Germany	Universitätsklinikum Frankfurt	Dr. med. Se-Jong You
Germany	Universitätsklinikum Frankfurt	Dr. med. Joachim Berkefeld
Germany	Universitätsklinikum Frankfurt	Dr. med. Fee Keil
Germany	Universitätsklinikum Frankfurt	Frau Heike Rai
Germany	Universitätsklinikum Frankfurt	Dr. med. Ferdinand Bohmann
Germany	Universitätsklinikum Frankfurt	Prof. Dr.med. Waltraud Pfeilschifter
Germany	Universitätsklinikum Frankfurt	Dr. med. Jan-Hendrik Schäfer
Germany	Universitätsklinikum Frankfurt	Dr. med. Gabriele Maurer
Germany	Universitätsklinikum Frankfurt	Prof. Dr. med. Christian Förch
Germany	Universitätsklinikum Frankfurt	Dr. med. Christoph Schmidt
Germany	Universitätsklinikum Frankfurt	Dr. med. Maximilian Rauch
Germany	Universitätsklinikum Frankfurt	Dr. med. Stella Breuer
Germany	Universitätsklinikum Frankfurt	Dr. med. Christoph Polkowski
Germany	Universitätsklinikum Frankfurt	Dr. med. Alexander Seiler
Germany	Universitätsklinikum Frankfurt	Dr. med. Nenad Polomac
Germany	Universitätsklinikum Frankfurt	Dr. med. Daniel Spitzer
Germany	Universitätsklinikum Frankfurt	Dr. med. Martin Schaller-Paule
Germany	Universitätsklinikum Frankfurt	Dr. med. Roxane-Isabelle Kestner
Germany	Universitätsklinikum Frankfurt	Dr. med. Erendira Gabriela Boss
Germany	Universitätsklinikum Frankfurt	Dr. med. Annemarie Brandhofe
Germany	Universitätsklinikum Frankfurt	Dr. med. Katharina Wenger-Alakmeh
Germany	Universitätsklinikum Frankfurt	Dr. med. Petar Trendafilov
Germany	Universitätsklinikum Frankfurt	Dr. med. Eike Steidl
Germany	Universitätsklinikum Frankfurt	Dr. med. Natalia Kurka
Germany	Universitätsklinikum Frankfurt	Dr. med. Sarah Gelhard
Germany	Universitätsklinikum Frankfurt	Dr. med. Monika Lauer
Germany	Universitätsklinikum Frankfurt	Dr. med. Jan-Erik Scholtz
Germany	Universitätsklinikum Frankfurt	Dr. med. Maya Hoelter
Germany	Universitätsklinikum Frankfurt	Dr. med. Sarah Reitz
Germany	Universitätsklinikum Frankfurt	Dr. med. Natalia Keeba
Germany	Universitätsklinikum Frankfurt	Dr. med. Katharina Gruber
Germany	Universitätsklinikum Frankfurt	Dr. med. Konstantin Kohlhase
Germany	Universitätsklinikum Frankfurt	Dr. med. Daniel Charisse
Germany	Universitätsklinikum Frankfurt	Dr. med. Samp Patrick
Germany	Universitätsklinikum Bonn	Prof Dr. Franziska Dorn
Germany	Universitätsklinikum Bonn	Dr. Nils Lehnen
Germany	Universitätsklinikum Bonn	Prof Dr Lazlo Solymosi
Germany	Universitätsklinikum Bonn	PD Dr. Daniel Paech
Germany	Universitätsklinikum Bonn	Prof Dr Gabor C. Petzold
Germany	Universitätsklinikum Bonn	Dr. Felix J. Bode
Germany	Universitätsklinikum Bonn	Dr. Julius N. Meissner
Germany	Universitätsklinikum Bonn	Dr. Taraneh Ebrahimi

Germany	Universitätsklinikum Bonn	Dr. Sebastian Stösser
Germany	Universitätsklinikum Bonn	Dr. Johannes M. Weller
Germany	Universitätsklinikum Bonn	Sara Theisen
Germany	Universitätsklinikum Bonn	Sandra Becker
Germany	Ev.-Luth. Diakonissenanstalt zu Flensburg	Prof. Dr.med. Stefan Müller-Hülsbeck
Germany	Ev.-Luth. Diakonissenanstalt zu Flensburg	Dr.med. Silke Hopf-Jensen
Germany	Ev.-Luth. Diakonissenanstalt zu Flensburg	Inga Petersen
Germany	Ev.-Luth. Diakonissenanstalt zu Flensburg	Andrea Merkle
Germany	Ev.-Luth. Diakonissenanstalt zu Flensburg	Petra Keese
Germany	Ev.-Luth. Diakonissenanstalt zu Flensburg	Dr.med. Birte Mesche
Germany	Ev.-Luth. Diakonissenanstalt zu Flensburg	Dr.med. Michael Preiß
Germany	Ev.-Luth. Diakonissenanstalt zu Flensburg	Dr.med. Maximilian Leißner
Germany	Ev.-Luth. Diakonissenanstalt zu Flensburg	Dr.med. Leonardo Marques
Germany	Klinikum Nürnberg Süd	Dr. med. Markus Holtmannspoetter
Germany	Klinikum Nürnberg Süd	Ms. Sabine Kohler
Germany	Klinikum Nürnberg Süd	Dr. med. Rüdiger Lange
Germany	Klinikum Nürnberg Süd	Dr. med. Heinz-Leonhard Voit-Höhne
Germany	Klinikum Nürnberg Süd	Dr. med. Florian Eff
Germany	Klinikum Nürnberg Süd	Dr. med. Michael Städt
Germany	Klinikum Nürnberg Süd	Ms. Romana Galczak
Germany	Klinikum Nürnberg Süd	Dr. med. Susanne Lamprecht
Germany	Klinikum Osnabrück	Dr.med. Lars Udo Krause
Germany	Klinikum Osnabrück	Sylvia Beer
Germany	Klinikum Osnabrück	Dr.med. Martina Petersen
Germany	Klinikum Osnabrück	Dr. med. Stephan Lowens
Germany	Klinikum Osnabrück	Dr.med. Christoph Kellinghaus
Germany	Klinikum Osnabrück	Dr.med. Sarah Strickmann
Germany	Otto-von-Guericke-University Magdeburg	Prof. Dr.med. Daniel Behme
Germany	Otto-von-Guericke-University Magdeburg	Prof. Dr.med. Michael Görtler
Germany	Otto-von-Guericke-University Magdeburg	Dr.med. Harald Paukisch
Germany	Otto-von-Guericke-University Magdeburg	Dr.med. Elie Diamandis
Germany	Otto-von-Guericke-University Magdeburg	Mostafa Ergawy
Germany	Otto-von-Guericke-University Magdeburg	Seraphine Zubel
Germany	Otto-von-Guericke-University Magdeburg	Dr.med. Thormann Maximilian
Germany	Otto-von-Guericke-University Magdeburg	Dr.med. Jens Neumann
Germany	Otto-von-Guericke-University Magdeburg	Dr.med. Maria Barleben
Germany	Otto-von-Guericke-University Magdeburg	Anne Assmann
Germany	Otto-von-Guericke-University Magdeburg	Ms. Hopp Katja
Germany	Otto-von-Guericke-University Magdeburg	Ms. Kollo Karen

Germany	Johannes Wesling Klinikum Minden	Prof. Dr.med. Jan Borggrefe
Germany	Johannes Wesling Klinikum Minden	Prof. Dr.med. Christoph Mönninghoff
Germany	Johannes Wesling Klinikum Minden	Dr.med. Michael Kuschnerow
Germany	Johannes Wesling Klinikum Minden	Dr.med. Jan Robert Kröger
Germany	Johannes Wesling Klinikum Minden	Dr.med. Thomas Röttcher
Germany	Johannes Wesling Klinikum Minden	Dr.med. Georg Reimann
Germany	Johannes Wesling Klinikum Minden	Dr.med. Claus Schneider
Germany	Johannes Wesling Klinikum Minden	Dr.med. Peter Schellinger
Germany	Johannes Wesling Klinikum Minden	Ms. Ramona Mücke
Germany	Johannes Wesling Klinikum Minden	Ms. Jasmin Retzlaff
Germany	Universitätsklinikum Leipzig AöR	Prof. Dr. med. Dominik Michalski
Germany	Universitätsklinikum Leipzig AöR	Prof. Dr. med. Karl-Titus Hoffmann
Germany	Universitätsklinikum Leipzig AöR	Dr. med. Johann Pelz
Germany	Universitätsklinikum Leipzig AöR	Priv.-Doz. Dr. med. Matthias Gawlitza
Germany	Universitätsklinikum Leipzig AöR	Rita Lachmund
Germany	Universitätsklinikum Leipzig AöR	Elfi Boxhammer
Norway	Oslo University Hospital	MD, PhD Anne Hege Aamodt
Norway	Oslo University Hospital	Study nurse Christina Kefalaykos
Norway	Oslo University Hospital	MD Bjørn Tennøe
Norway	Oslo University Hospital	Prof. Mona Skjelland
Norway	Oslo University Hospital	Dr., MD Brian Enriquez
Norway	Oslo University Hospital	Dr., MD Stephen Ryan
Norway	Oslo University Hospital	Dr., MD Jon-Anders Tunold
Norway	Oslo University Hospital	Dr., MD Erika Hallerstig
Norway	Oslo University Hospital	Dr., MD Jogrim Søyland
Norway	Oslo University Hospital	Dr., MD Simen Sandvik
Norway	Oslo University Hospital	Dr., MD Sjur Prestsæter
Norway	Oslo University Hospital	Dr., MD Jesper Sømark
Norway	Oslo University Hospital	Dr. MD Maria Argren
Norway	Oslo University Hospital	Dr. MD PhD Karolina Skagen
Norway	Oslo University Hospital	Dr. MD Lars Tveit
Norway	Haukeland University Hospital, Bergen	Annette Fromm, MD PhD MSc
Norway	Haukeland University Hospital, Bergen	Gaute Kjellevold Wathle MD
Norway	Haukeland University Hospital, Bergen	Anne Margrethe Kaalaas Størda
Slovakia	Jessenius Faculty of Medicine in Martin	Assoc. prof., MD, PhD Kamil Zelenak
Slovakia	Jessenius Faculty of Medicine in Martin	MD, PhD Martin VORČÁK
Slovakia	Jessenius Faculty of Medicine in Martin	MD Jan SÝKORA
Slovakia	Jessenius Faculty of Medicine in Martin	MD Adam KRKOŠKA
Slovakia	Jessenius Faculty of Medicine in Martin	MD Jozef SIVÁK
Slovakia	Jessenius Faculty of Medicine in Martin	MD Jakub SORŠÁK

Slovakia	Jessenius Faculty of Medicine in Martin	Prof. MD, PhD Egon KURČA
Slovakia	Jessenius Faculty of Medicine in Martin	MD Jana ZELEŇÁKOVÁ
Slovakia	Jessenius Faculty of Medicine in Martin	Assoc. Prof., MD, PhD Vladimír NOSÁĽ
Slovakia	Faculty Hospital Trnava	Ph.D., MPH, EBIR Andrej Klepanec
Slovakia	Faculty Hospital Trnava	M.D. Jozef Haring
Slovakia	Faculty Hospital Trnava	M.D., Ph.D. Georgi Krastev
Slovakia	Faculty Hospital Trnava	M.D. Juraj Cisár
Slovakia	Faculty Hospital Trnava	M.D. Martin Daniš
Slovakia	Faculty Hospital Trnava	M.D. Ján Haršány
Slovakia	Faculty Hospital Trnava	M.D. Xénia Bavúzová
Slovakia	Faculty Hospital Trnava	M.D. Marián Ondrejkovič
Spain	La Paz University Hospital	Dr. Blanca Fuentes Gimeno
Spain	La Paz University Hospital	Dr. Alberto Álvarez
Spain	La Paz University Hospital	Dr. Andrés Barrios
Spain	La Paz University Hospital	Dr. Andrés Fernández
Spain	La Paz University Hospital	Dr. Elena de Celis
Spain	La Paz University Hospital	Dr. Gerardo Ruiz
Spain	La Paz University Hospital	Dr. Jorge Rodríguez
Spain	La Paz University Hospital	Dr. Laura Casado
Spain	La Paz University Hospital	Dr. Mª Alonso de Leciñana
Spain	La Paz University Hospital	Dr. Pedro Navia
Spain	La Paz University Hospital	Dr. Ricardo Rigual
Spain	La Paz University Hospital	Victoria Hernández
Spain	La Paz University Hospital	Rocio Prieto-Pérez
Spain	La Paz University Hospital	Carlota Rivera-Bengoa
Switzerland	University Hospital Basel	Prof. Dr. Marios Psychogios
Switzerland	University Hospital Basel	Dr. Ioannis Tsogkas
Switzerland	University Hospital Basel	Dr. Alex Brehm
Switzerland	University Hospital Basel	Dr. Anh Nguyễn
Switzerland	University Hospital Basel	Dr. Lukas Nussbaum
Switzerland	University Hospital Basel	Dr. Michail Panagiotis Giannakakis
Switzerland	University Hospital Basel	Dr. Nikos Ntoulias
Switzerland	University Hospital Basel	PD Dr. Peter Sporns
Switzerland	University Hospital Basel	Prof. Dr. Urs Fischer
Switzerland	University Hospital Basel	Michele Damaskinos
